# The E-cadherin repressor slug and progression of human extrahepatic hilar cholangiocarcinoma

**DOI:** 10.1186/1756-9966-29-88

**Published:** 2010-07-01

**Authors:** Ke-jun Zhang, Dong-sheng Wang, Shao-yan Zhang, Xue-long Jiao, Chun-wei Li, Xin-sheng Wang, Qin-chao Yu, Hai-ning Cui

**Affiliations:** 1 General Surgery, the Affiliated Hospital of medical College, Qingdao University, Qing dao, Shandong Province. 266003. China; 2 Laboratorian, the Affiliated Hospital of medical College, Qingdao University, Qing dao, Shandong Province. 266003. China; 3 Urinary surgery, the Affiliated Hospital of medical College, Qingdao University, Qing dao, Shandong Province. 266003. China; 4 Anatomic Pathology, the Affiliated Hospital of Hainan Medical college, Haikou. 527010. China

## Abstract

**Objectives:**

This study explored the expression and function of Slug in human extrahepatic hilar cholangiocarcinoma (EHC) to identify its role in tumor progression.

**Methods:**

The expression of Snail and Slug mRNA in 52 human tissue samples of EHC was investigated. The mRNA of Snail and Slug were quantified using reverse transcriptase-PCR, and correlations with E-cadherin expression and clinicopathological factors were investigated. We then investigated transfection of Slug cDNA in endogenous E-cadherin-positive human EHC FRH0201 cells, selectively induced the loss of E-cadherin protein expression, and then small interfering RNA (siRNA) for inhibition of Slug expression in endogenous Slug-positive human EHC QBC939 cells, selectively induced the loss of Slug protein expression. A Boyden chamber transwell assay was used for invasion.

**Results:**

Slug mRNA was overexpressed in 18 cases (34.6%) of EHC compared with adjacent noncancerous tissue. E-Cadherin protein expression determined in the same 52 cases by immunohistochemistry was significantly down-regulated in those cases with Slug mRNA overexpression (P = 0.0001). The tumor and nontumor ratio of Slug mRNA was correlated with nodal metastasis(p = 0.0102), distant metastasis (p = 0.0001)and Survival time(p = 0.0443). However, Snail mRNA correlated with neither E-cadherin expression nor tumor invasiveness. By inhibiting Slug expression by RNA interference, we found that reduced Slug levels upregulated E-cadherin and decreased invasion in QBC939 cell. When the QBC939 cells was infected with Slug cDNA,, significant E-cadherin was downregulated and increased invasion in QBC939 cell.

**Conclusions:**

The results suggested that Slug expression plays an important role in both the regulation of E-cadherin expression and in the acquisition of invasive potential in human EHC. Slug is possibly a potential target for an antitumor therapy blocking the functions of invasion and metastasis in human EHCs.

## Introduction

Cholangiocarcinoma is a cancer arising from bile duct epithelium. It is one of the most difficult diseases to treat. Three-year survival rates of 35 to 50% can be achieved in only a few numbers of patients when negative histological margins are attained at the time of surgery [1]. The reason for this poor prognosis is that cholangiocarcinoma exhibits extensive local invasion and frequent regional lymph node metastasis[2]. but the mechanisms through which Cholangiocarcinoma acquires such invasive potentials are not well understood.

E-Cadherin-mediated cell-to-cell adhesion plays a critical role in the maintenance of cell polarity and environment [3] . E-Cadherin was reported to be down-regulated and closely related to tumor invasion and metastasis in many cancers[4-6] . Genetic and epigenetic alteration of E-cadherin was also reported [3] . Somatic mutation, loss of heterozygosity of the *E-cadherin *gene, and CpG methylation around the promoter region of the *E-cadherin *gene were noted in human gastric cancer, breast cancer, and Hepatocarcinoma[7-11]. However, E-cadherin promoter hypermethylation is not always associated with loss of expression [11], and evidence has been presented that E-cadherin expression could be repressed by mechanisms other than promoter hypermethylation [8] . The heterogeneity and reversibility of E-cadherin protein expression are both controversial areas [3]. Recently, the Slug transcription factor was reported to directly repress E-cadherin expression in many epithelial cancers associated with epithelial-mesenchymal transitions [12] . Reverse correlation of Slug and E-cadherin expression has been noted in many malignant cells[13-19]. It has reported that Snail, a zing-finger protein, is a likely repressor of E-cadherin in carcinoma Cells[20-22]. However, we can find no documentation regarding the expression of Snail or Slug in human EHC tissue. In this study, we investigated whether Slug represses E-cadherin expression in human EHC cells. The levels of expression a of Snail and Slug mRNA were detected in a series of human EHC samples, and correlations between Snail/Slug expression and clinicopathological factors were analyzed. Our evidence suggests that Slug, rather than Snail, may contribute to both E-cadherin expression and to the progression of EHCs.

## Materials and methods

### Patients

This present retrospective study was based on data obtained using surgically resected tissues from 52 consecutive Chinese patients who underwent hepatectomy for EHCs. Written informed consent was obtained from each patient before tissue acquisition. All data were collected in the Department of Anatomical Pathology, Afflited hospital of Qingdao medical college, Qingdao university (Qingdao, China) from July 2000 to Sep. 2008. All tumors were defined as EHC, and pathological features of the tumors were determined histologically based on classifications of the Liver Cancer Study Group of China . Histological grades of the tumors consisting of more than two features were defined by the most prominent feature, and those components were selected for immunohistochemical studies.

### Real-Time Quantitative RT-PCR of Snail and Slug

Total RNA was extracted and purified from 52 paired samples of fresh frozen cancerous tissues and noncancerous bile tissues using Trizol Reagent (Life Technologies, Inc.) according to the manufacturer's instructions. For reverse transcriptase reaction, we used 5 μg of the RNA, random hexamers, and Superscript II reverse transcriptase (Life Technologies, Inc.) according to the manufacturer's instructions. The oligonucleotide primers and TaqMan probes designed for Snail and Slug were as follows: Snail (5'-ACCACTATGCCGCGCTCTT-3' and 5'-GGTCGTAGGGCTGCTGGAA-3'); Slug (5'-TGTTGCAGTGAGGGCAAGAA-3' and 5'-GACCCTGGTTGCTTCAAGGA3'); and TaqMan probe (Snail, 5'-6FAM-TCGTCAGGAAGCCCTCCGACCC-TAMRA-3' and Slug, 5'-6FAM-AGGCTTCTCCCCCGTGTGAGTTCTAATG-TAMRA-3'). Each primer was placed in a different exon to avoid amplification of contaminating genomic DNA. Primers and probe for GAPDH (TaqMan GAPDH control reagent kit) were purchased from Perkin-Elmer Applied Biosystems (Foster City, CA). Real-time quantitative PCR was done using the ABI Prism 7700 Sequence Detection System (Perkin-Elmer Applied Biosystems), as described above. Real-time PCR assays were done in triplicate, and the mean values were used for calculations of mRNA expression. Finally, the Snail and Slug mRNA expression ratios for tumorous (T) and nontumorous (N) tissues were calculated as follows: *R *= [Snail or Slug (T)/GAPDH (T)]/[Snail or Slug (N)/GAPDH (N)] × 102. Cases were designated as either overexpression (*R *> 100) or nonoverexpression (*R *≤ 100) cases.

### Immunohistochemical Staining of E-Cadherin

Formalin-fixed, paraffin-embedded tissue sections from 52 EHC cases that corresponded to the RNA extracted cases were processed for immunohistochemical staining, as described previously [23] . A primary monoclonal Ab against E-cadherin (diluted 1:1000; Transduction Laboratories) was used. Positive immunoreactivity of normal bile duct epithelium was confirmed as a positive control for each specimen [24] . Immunohistochemical staining was examined under a light microscope by two pathologists. The cell staining of E-cadherin was evaluated semiquantitatively, and tumors were divided into two groups: (*a*) preserved pattern: >75% of tumor cells staining and (*b*) reduced pattern: <75% of tumor cells staining, as described elsewhere [23] .

### Real-time RT-PCR for E-cadherin mRNA and Slug mRNA in EHC cell lines

QBC939, SK-Ch-1, FRH 0201 cells, the cultured human EHC cell line, were supplied from Cell Resource Center, FUDAN University (Shanghai, China). These cells were cultured at 37°C in 5% CO2 in RPMI 1640, containing 10% FBS. Upon reaching 70% confluence cells were lysed into Trizol reagent (Gibco, UK) for mRNA extraction and evaluation of E-cadherin mRNA and Slug mRNA expression by Real-time quantitative RT-PCR. Real-time quantitative PCR was done using the ABI Prism 7700 Sequence Detection System (Perkin-Elmer Applied Biosystems) as described previously [23]. Briefly, each PCR mixture contained 1 μl of cDNA, TaqMan Universal PCR master mix (Perkin-Elmer Applied Biosystems), primer pair, and TaqMan probe in a final volume of 50 μl. The PCR conditions were an initial denaturation step of 2 min at 50°C and 10 min at 95°C, followed by 40 cycles consisting of 15 s at 95°C, and a 1 min at 60°C. Serial 1:10 dilutions of plasmid DNA were analyzed for each target cDNA, and these served as standard curves from which we determined the rate of change of threshold cycle values. The amount of target gene expression was calculated from the standard curve, and quantitative normalization of Slug cDNA in each sample was done using GAPDH as an internal control.

### Subcloning of Human Slug cDNA and Construction of Expression Plasmids

The full coding region of human *Slug *was amplified by PCR using primers (5'-GCTGTAGGAACCGCCGTGTC-3' and 5'-ATTTGTCATTTGGCTTCGGAGTG-3') from cDNA of human EHC, and the product was cloned into the pT7 Blue vector (Novagen, Madison, WI). Isolated DNA sequences were determined using a cycle sequencing procedure. Slug cDNA was then subcloned into the bicistronic expression vector pGEM-T -EGFP (Clontech, Palo Alto, CA), which allows for translation of both the genes of interest and the EGFP.

### Cell Culture and Transient Transfection of Slug cDNA

FRH 0201 cells were cultured at 37°C in 5% CO2 in RPMI 1640 (Life Technologies, Inc., Rockville, MD), containing 10% FBS (Life Technologies, Inc.). FRH 0201 cells (1 × 106) were grown in 3.5-cm dishes and transiently transfected with 2 μg of the pSlug-EGFP plasmid, as well as the empty pEGFP (mock) plasmid using Lipofectamine (Life Technologies, Inc.), according to the manufacturer's instructions. At 48 h after transient transfection, Slug siRNA-transfected cells, which expressed both Slug and EGFP, were confirmed by epiluminescence fluorescence microscopy (Axioscop2, Zeiss, Germany) .

### Small interfering RNA (siRNA) for inhibition of slug expression

Three stealth small interfering RNA (siRNA) duplex oligoribonucleotides specific for Slug were synthesized. The sequences were as follows:

1) sense 5'-UUAACAGCAAACUCAGUUGAAAUGG-3',

antisense 5'-CCAUUUCAACUGAGUUUGCUGUUAA-3';

2) sense 5'-UGAAUUAGGAAACUGAUCUUCCGGA-3',

antisense 5'-UCCAGAAGAUC AGUUUCCU AAUUCA-3';

3) sense 5'-AAAUCUUUCAUGAUGAUUCCCUCGG-3',

antisense 5'- CCGAGGGAAUCAUGAAAGAUU U-3'.

siRNA oligos were transfected into cholangiocarcinoma cells by using BLOCK-iT transfection kit (Invitrogen, Carlsbad, CA) according to the manufacturer's protocol. The BLOCK-iT fluorescent oligo that is not homologous to any known genes was used as transfection efficiency detector and a negative control to ensure against induction of non-specific cellular events caused by introduction of the oligo into cells. Among the three siRNA oligo duplexes specific for slug, the one that required the smallest concentration to achieve the desired knockdown effect was selected and used in all experiments.

### Real-time RT-PCR for E-cadherin mRNA after transient transfection of Slug siRNA

siRNA oligos were transfected into QBC939 (the highest level of Slug expression) cells (2 × 105) by using BLOCK-iT transfection kit (Invitrogen, Carlsbad, CA) according to the manufacturer's protocol for 48 h. The mRNA inhibiting levels were assayed with Real-time RT-PCR .

### Tumor invasion in Matrigel-coated chambers

To determine invasive ability, siRNA-Slug , Slug cDNA or mock control cells (1.25 × 105 per well)were plated on the BD Matrigel invasion chambers (BD Biosciences). Medium in the upper chamber was supplemented with 5% FCS. In the lower chamber, FCS concentration was 10%. After 24 h, cells migrated into the lower chamber were stained and counted. Experiments were carried out in triplicate and repeated twice.

### Statistical Analysis

Follow-up was obtained through office records, telephone contact, or E-mail. Patient follow-up was complete up to September, 2008. Survival was calculated from the date of resection to one year after postoperation. All results were expressed as mean ± SE. Comparisons between Snail/Slug expression levels (R; > 100 or ≤ 100) and E-cadherin expression patterns were evaluated using χ^2 ^test, and comparisons between the Snail/Slug expression ratios and clinicopathological parameters were evaluated using *t *test or *F *test. *P *of < 0.05 was considered to have statistical significance.

## Results

### Expression of Slug and Snail mRNA in extrahepatic hilar cholangiocarcinoma

We quantified the copy numbers of Slug and Snail mRNA in 52 pairs of EHC tissue and noncancerous bile duct tissues using a TaqMan probe on ABI Prism 7700 Sequence Detection System, as described above. The copy number of Slug, Snail and GAPDH mRNA ranged from 218.4 to 83096, 117.8 to 15262, and 1238.56 to 6287429, respectively. Slug and Snail expression were standardized using the expression of the GAPDH housekeeping gene as the internal control. The cancerous (T)/noncancerous (N) ratio of mRNA (R) was then calculated to determine Snail and Slug mRNA levels in each case. Slug mRNA levels in cancerous tissue ranged from 0.823 to 58.9 (mean ± SE: 13.8 ± 3.1) and that of noncancerous tissue from 4.14 to 142 (mean ± SE: 39.6 ± 4.8). The ratio (R) of Slug ranged from 0.04 to 658 (mean ± SE: 63.4 ± 19.3). 18 (34.6%) of 52 examined samples were defined as cases overexpressing Slug mRNA. The Snail mRNA levels were from 2.18 to 342 (mean ± SE: 47.8 ± 13.02) in cancerous tissue and from 8.80 to 163 (mean ± SE: 62.45 ± 6.8) in noncancerous tissue. The ratios (R) of Slug ranged from 3.14 to 1049 (mean ± SE: 132 ± 38.6). 12 (23%) of 52 samples examined were defined as cases overexpressing Snail mRNA.

### Relationship between Slug and Snail expression and clinicopathologic data

The relationship between Slug and Snail expression and clinicopathologic features is summarized in Table 1. The mean Slug mRNA ratio was significantly higher in cases of nodal metastasis (59.8 *versus *77.4, *P *= 0.0102)and distant metastasis (64.8 *versus *146.3, *P *= 0.0001). Patients with increased Slug mRNA(9/52)survived significantly shorter than those with reduced Slug mRNA expression (43/52) (P = 0.0443). Cases of lymphatic invasion and perineural invasion also had high Slug mRNA ratios compared with the cases without invasion, although there was no statistical significance because of the distribution of the ratio [76.5 *versus *68.3 (*P *= 0.1404), 60.4 *versus *54.9 (*P *= 0.134), respectively. There was no statistical significance of Snail expression on clinicopathological parameters.

**Table 1:** Comparison of clinicopathological variables dependent on Snail and Slug mRNA ratios

	Slug mRNA (mean ± SE)	P	Snail mRNA (mean ± SE)	P
mean age (yr)				
<65(15)	86.9 ± 25.5		149.3 ± 57.4	
>65(37)	78.3 ± 19.7	0.1969	171.2 ± 62.8	0.249
Gender				
62.2 ± 32.3	62.2 ± 32.3		127.4 ± 35.6	
70.6 ± 17.5	70.6 ± 17.5	0.2415	124.3 ± 71.8	0.8488
Histologic grading				
G1 (29)	66.4 ± 13.6		107.2 ± 60.2	
G2 (16)	58.0 ± 26.56		114.7 ± 53.5	
G3 (7)	73.2 ± 33.8	0.2523	125.4 ± 41.4	0.7252
Histology				
Well(13)	69.2 ± 18.4		95.7 ± 28.3	
Mod.(27)	76.0 ± 15.8		108.4 ± 46.5	
Poor(12)	85.6 ± 29.2	0.135	100.7 ± 31.1	0.6109
Depth of invasion				
T1(8)	79.2 ± 12.4		117.1 ± 28.0	
T2(32)	68.4 ± 19.7		98.4 ± 34.6	
T3(12)	80.2 ± 30.5	0.1962	109 ± 36.3	0.3260
Surgical margin involvement				
Negative (n = 38)	66.4 ± 16.7		102.6 ± 49.4	
Positive (n = 14)	77.6 ± 31.5	0.2277	124.8 ± 60.0	0.197
Nodal metastasis				
Negative (n = 32)	**59.8 ± 23.3**		86.8 ± 75.6	
Positive (n = 20)	**77.4 ± 22.8**	0.0102	109.8 ± 35.2	0.1448
Lymphatic invasion				
Negative (n = 10)	**68.3 **± 10.9		180.3 ± 49.4	
Positive (n = 42)	75.6 **± **16.4	0.1404	154. 5 ± 40.1	0.0865
Venous invasion				
Negative (n = 15)	79.6 ± 30.7		120 ± 121.7	
Positive (n = 37)	87.2 ± 24.6	0.3524	134.5 ± 30.6	0.1015
Perineural invasion				
Negative (n = 12)	60.4 ± 16.8		155.2 ± 26.2	
Positive (n = 40)	52.9 ± 14.4	0.134	166.3 ± 40.4	0.3758
Distant metastasis				
Negative (n = 44)	64.8 ± 19.6		163.8 ± 13.6	
Positive (n = 8)	146.3 ± 33.2	0.0001	143.3 ± 27.5	0.0747
Survival (mo)				
<12 (n = 9)	126.8 ± 24.5		176.5 ± 87.2	
>12 (n = 43)	103.3 ± 36.7	0.0443	163.4 ± 54.4	0.5596

Among the 18 Slug overexpression cases, 13 cases (72.2%) showed portal vein invasion and 7 (38.9%) showed liver artery invasion, whereas there were only 7 (20.6%)with portal vein invasion and 2 (5.9%) with liver artery invasion in 34 cases of Slug nonoverexpression.

In addition, 10/18 showed remarkably high Slug mRNA levels (*R *> 200), and these were all with portal vein invasion.

### E-cadherin protein expression in EHC samples with or without Snail/Slug mRNA overexpression

**Figure 1:**
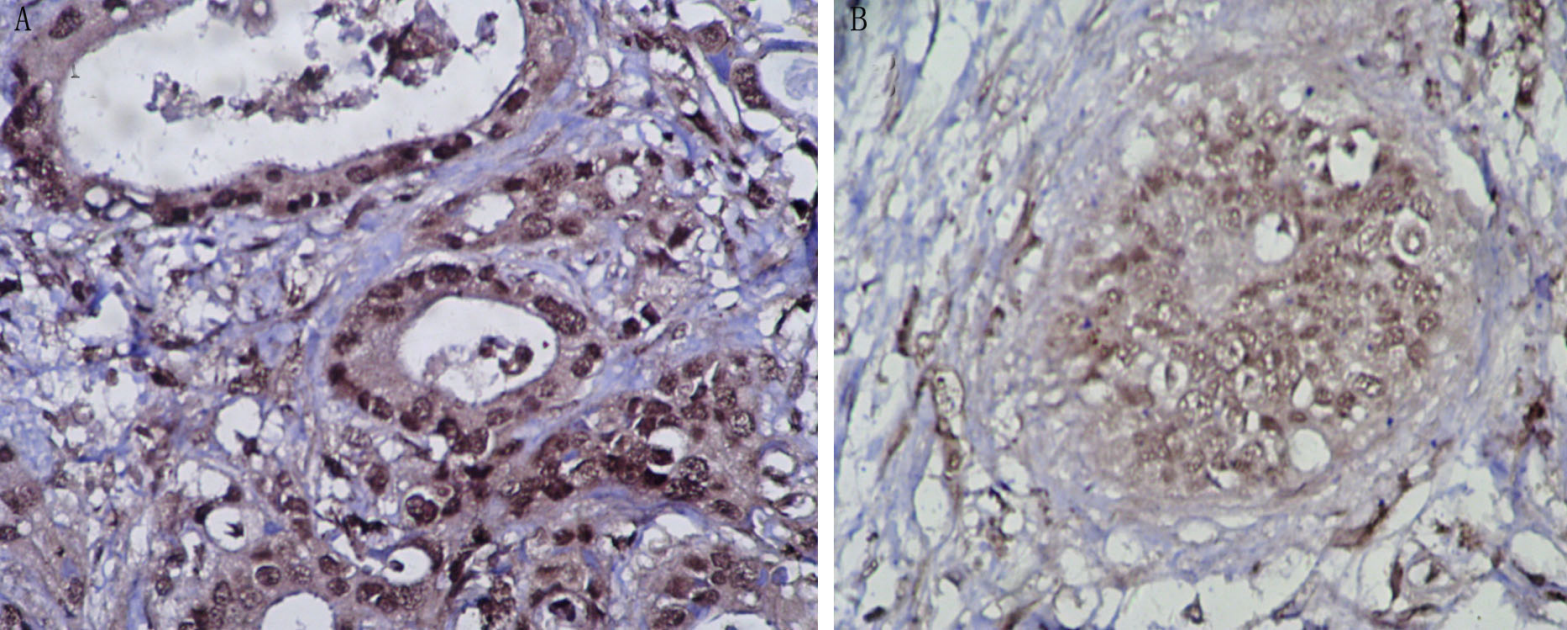
**Representative example of the E-cadherin expression determined by immunohistochemistry**. **A**, carcinoma cells showed strong expression (preserved pattern) in the Slug nonoverexpression case. **B**, carcinoma cells showed weak expression (reduced pattern) in the Slug overexpression case. (magnification, ×400).

Expression of E-cadherin protein was also analyzed immunohistochemically. E-cadherin was expressed in membrane and/or cytoplasm.19 of 52 EHCs (36.5%) had a reduced expression pattern (Fig. 1). These findings did not significantly correlate with clinicopathological features such as distant metastasis, portal vein invasion, and liver artery invasion. The relationship between Snail/Slug mRNA expression and E-cadherin protein expression patterns was then determined in the EHC samples. Slug mRNA overexpression significantly correlated with E-cadherin reduced expression (Table 2) . 13 (72.2%) of 18 cases overexpressing Slug showed a reduced E-cadherin expression pattern, whereas only 6 of 34 cases of Slug nonoverexpression (17.6%) had a reduced pattern, with a statistically significant difference (*P *= 0.0001). However, there was no significant correlation between Snail overexpression and E-cadherin expression (Table 2)

**Table 2:** Comparison of Snail and Slug expression between preserved and reduced patterns of E-cadherin

	E-cadherin expression Preserved (*n *= 33)	E-cadherin expression Reduced (*n *= 19)	*P*
Slug mRNA			
Overexpression (*n *= 18)	5 (27.8)	13 (72.2)	
Nonoverexpression (*n *= 34)	28 (82.4)	6 (17.6)	0.0001
Snail mRNA			
Overexpression (*n *= 12)	7 (58.3)	5(41.7)	
Nonoverexpression (*n *= 40)	26 (65)	14(35)	0.9993

### Ectopic expression of Slug to down-regulate E-Cadherin expression in EHC cell lines

**Figure 2:**
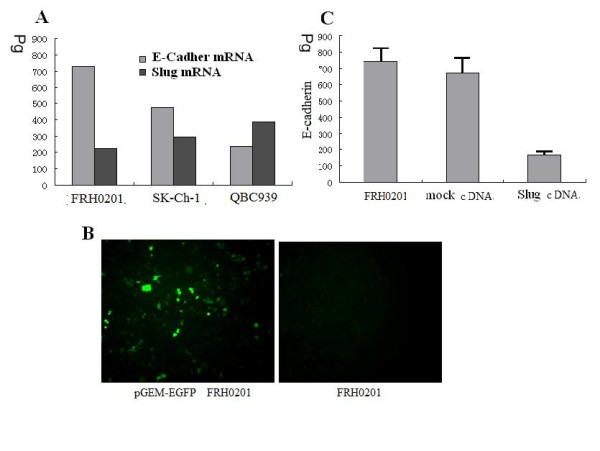
**A Expression of E-Cadher mRNA in QBC939, SK-Ch-1, FRH 0201 cells**. In vitro cleavage effect of different ribozymes on E-Cadherin mRNA and Slug mRNA. The reaction product of in vitro ribozyme cleavage was analyzed by absolute real-time quantitative PCR. The amplification plots and standard curve were obtained with the in vitro transcript from E-Cadherin. Serial 10-fold dilutions with 9 × 108 to 9 × 10-2 pg per reaction well were made in EASY Dilution (Takara). Amplification was repeated three times for each dilution. **It showed **the cell line FRH 0201 had the highest expression of E-Cadherin m RNA and the lowest expression of Slug mRNA. **B **Evaluation of transfection efficiencies. It showed the transfection efficiency was 43.6% 48 h after Slug transfection. **C **E-cadherin in Slug transfected and mock-transfected FRH 0201 cells. In vitro cleavage effect of different ribozymes on E-Cadherin mRNA. The reaction product of in vitro ribozyme cleavage was analyzed by absolute real-time quantitative PCR. The amplification plots and standard curve were obtained with the in vitro transcript from E-Cadherin. Serial 10-fold dilutions with 9 × 108 to 9 × 10-2 pg per reaction well were made in EASY Dilution (Takara). Amplification was repeated three times for each dilution. It showed Slug overexpression repressed E-cadherin expression in FRH 0201.

E-Cadherin mRNA expression was examined in a panel of three cholangiocarcinoma cell lines QBC939, SK-Ch-1, FRH 0201 by real-time PCR and results showed that the cell line FRH 0201 had the highest expression level of E-Cadherin mRNA and the lowest expression of Slug mRNA (Fig 2A). In this regard, the cell line FRH 0201 was chosen for *the *studies..

The cell line FRH 0201 was transiently transfected with either full length human Slug cDNA-GFP vector or the control empty GFP vector. 48 h after transfection, cells were lysed and processed for mRNA analysis. In Fig 2B, the green fluorescent color indicates FRH 0201 cells transfected with control empty GFP vector. Cells were counted on the photographs and the ratio between green fluorescent cells and total cell number was taken as transfection efficiency. The transfection efficiency was 43.6% 48 h after transfection.

Slug transfectants showed a remarkably reduced expression of E-cadherin protein, whereas positive E-cadherin expression was observed in nontransfected FRH 0201 cells. On the other hand, E-cadherin expression was homogeneously preserved in mock-transfected cells (Fig 2C). These observations provided direct evidence that Slug repressed E-cadherin expression in human cholangiocarcinoma cells.

### siRNA Slug increases E-cadherin expression

**Figure 3:**
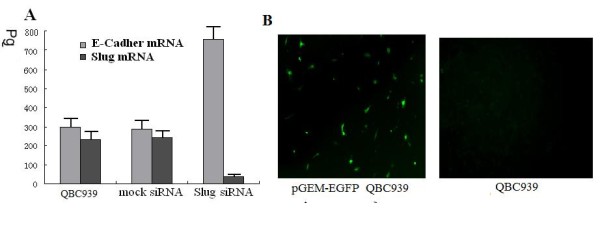
**A Expression of E-cadherin in QBC939 cells**. The reaction product of in vitro ribozyme cleavage was analyzed by absolute real-time quantitative PCR. The amplification plots and standard curve were obtained with the in vitro transcript from E-Cadherin. Serial 10-fold dilutions with 9 × 108 to 9 × 10-2 pg per reaction well were made in EASY Dilution (Takara). Amplification was repeated three times for each dilution. It showed Slug inhibition increased E-cadherin expression in QBC939 cells. **B **Evaluation of transfection efficiencies. It showed the transfection efficiency was 31.4% 48 h after siRNA-Slug transfection.

**Slug **mRNA expression was examined in a panel of three cholangiocarcinoma cell lines QBC939, SK-Ch-1, FRH 0201 by real-time PCR and results showed that the cell line QBC939 had the highest expression level of **Slug **mRNA (Fig 3A). In this regard, the cell line QBC939 was chosen for *the *studies. The cell line QBC939 was transiently transfected with Slug siRNA oligos for 48 h by using BLOCK-iT transfection kit. Cells were lysed and processed for mRNA analysis. The transfection efficiency was 32.4% 48 h after transfection (Fig 3B). siRNA-Slug transfectants showed a remarkably increased expression of E-cadherin. (Fig 3A). The observations provided direct evidence that Slug inhibition increased E-cadherin expression in human cholangiocarcinoma cells.

### Cell invasion detection

**Figure 4:**
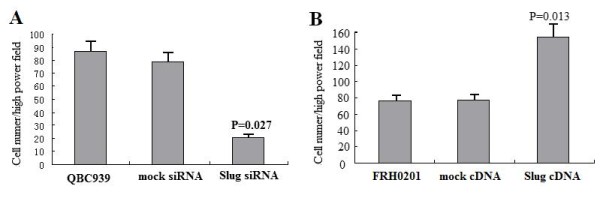
**siRNA knockdown of Slug and overexpression of Slug with the invasive potential in EHC cells. **Cells were seeded in the upper chamber in medium supplemented with 5% FCS. Results are reported as percent migration ± SD compared with untreated cells. Experiments were carried out twice in triplicate. A Slug silencing inhibits invasion potention of QBC939 cells in Matrigel-coated invasion chambers. B Slug overexpression promotes invasive potential in FRH 0201 cells in Matrigel-coated invasion chambers.

We tested whether Slug knockdown affected the invasion capabilities of QBC939 cells by using an *in vitro *invasion assay. Cells were seeded in the upper part of a Matrigel-coated invasion chamber in a reduced (5%) FCS concentration. After 24 h, cells that migrated in the lower chamber containing a higher (10%) FCS concentration were stained and counted. In Slug-silenced cell lines, invasion was significantly reduced (Fig. 4A; P < 0.05). Compared with untreated cells, or mock-siRNA cells, no further decrease in invasion was observed .

We also tested the effects of Slug overexpression on the invasion capability of FRH 0201 cells. Compared with data obtained using the parental cell lines, Slug cDNA-treated FRH 0201 cells exhibited increased invasion (Fig. 4B; P < 0.05). Together, these data show that Slug modulates invasion of EHC cells *in vitro*.

## Discussion

Recent direct evidence shows that Snail transcription factor and its family protein Slug repress E-cadherin expression in human cancer cell lines[13,22,25-30] . Down-regulation of E-cadherin causes loss of cell-to-cell adhesion. Impaired adhesion characterizes the potential of invasion and metastases, crucial steps for progression of hepatocarcinoma[3]. Thus, the down-regulation of E-cadherin promotes invasion and metastases of hepatocarcinoma and *vice versa *[31] . To confirm the function of Slug in EHC, we used E-cadherin-positive FRH0201 cells and slug positive QBC939 cells reported above that E-cadherin and Slug inversely express in FRH0201 and QBC939 cell lines.

Our data revealed direct evidence that transient Slug expression can suppress E-cadherin protein expression and increased the motility and invision potential in QBC939 cells. Transient Slug inhibition can increase E-cadherin protein expression in FRH0201 cells, and decreased the motility and invision potential.

We investigated Slug mRNA using RT-PCR and confirmed that Slug mRNA is expressed in EHC samples. We then quantitatively analyzed the mRNA expression levels of Slug in both cancerous and noncancerous tissues of EHCs. We used the cancerous/noncancerous ratio of Slug mRNA to evaluate Slug expression levels in each case. 18 (34.6%) were determined to be Slug overexpression cases, and this overexpression significantly correlated with reduced E-cadherin expression. Our data show that Slug, rather than Snail, functions as a suppresser of E-cadherin in human EHC tissue, as well as in cultured EHC cells. Recently, Paras *et al*. [18] reported that Slug contributed to the down-regulation of E-cadherin expression in esophageal adenocarcinoma lines. Although both proteins are produced in all vertebrate species, their functions are different among various species and different cells [32,33]. These data suggest that E-cadherin production of carcinoma cells should be regulated by the different transcriptional repressors among the different cells or tissues.

We found significant E-cadherin reduction in Slug overexpression cases, however, there were 28 (82.4%) with reduced E-cadherin expression but without Slug overexpression. Kanai *et al.*[34] reported that 48% show DNA hypermethylation of the E-cadherin promoter region and 42% show loss of heterozygosity at the locus adjacent to the E-cadherin gene in HCC. Genetic mutation of the E-cadherin gene was detected in breast, gastric, and gynecological cancers, which showed a uniform loss of E-cadherin expression[35-37] . To date, a genetic mutation of the E-cadherin gene has not been reported in cases of EHC in which loss of E-cadherin expression is considered to be heterogeneous and reversible . Therefore, E-cadherin expression in EHC may be regulated not just by the Slug transcriptional factor but also by other genetic and/or epigenetic alterations such as DNA mutation and/or methylation. Additional studies are required to reveal the entire regulatory mechanism of E-cadherin expression in EHC tumors.

In this study, Slug mRNA overexpression correlated with metabasis and invasion of surgically resected human EHC. High expression of Slug mRNA has significantly shorter survival, the expression of Slug mRNA in EHC is an independent poor prognostic factor. EHC is hence a useful marker for predicting the outcome of patients with EHC who had a surgical resection of the tumor. Our data show that Slug, rather than Snail, negatively regulates E-cadherin expression, but it may also regulate the expression of other genes involved in the invasive potential of EHC. E-Cadherin has been reported to involve in tumor invasiveness [38-42] , but the relationships between E-cadherin and clinicopathological factors were not consistent among these studies. In this study, E-cadherin was not found to be related to any clinicopathological factors. Differences of etiology and methods of evaluation might cause this discrepancy [40-42] . Additionally, the reversibility of E-cadherin expression should be considered. Slug and other family proteins bind to specific target genes and function as transcriptional repressors, but it is considered that the repression of E-cadherin alone is not sufficient to explain the role of Slug in cell migration and cancer development. The possible involvement of rhoA, rhoB, and other molecules, as well as E-cadherin, in the Slug pathway that controls cell motility has been considered for *Drosophila*, *Caenorhabditis elegans*, and vertebrate[43-46]. Additional investigations are needed to fully understand the functions and target genes of Slug protein in EHCs.

## Competing interests

The authors declare that they have no competing interests.

## Authors' contributions

ZKJ, WDS and ZSY designed the experiments. ZKJ and JXL carried out most of experiments and drafted the manuscript. WXS, YQC and CHN carried out the immunohischemistry and RT-PCR. LCW and WDS participated in statistical analysis and and interpretation of data. All authors read and approved the final manuscript.
